# Supplementary material for: Chromosome-level genome assembly of a doubled haploid brook trout (Salvelinus fontinalis)
Source: G3 (Bethesda). 2025 Mar 25;15(6):jkaf066. doi: 10.1093/g3journal/jkaf066 (PMC12134987; doi:10.1093/g3journal/jkaf066)
Supplement: jkaf066_Supplementary_Data [file jkaf066_supplementary_data.zip › Supplementary_Material_Legends_G3-2024-405170.docx]

**Supplementary figures**

**Figure S1.** Comparison of brook trout (*Salvelinus fontinalis*) linkage maps produced in the current study (16,203 markers; *x*-axis) and in Sutherland et al. (2016) (3,826 markers; *y*-axis). Both maps relied on RADseq data from Sutherland et al., but were produced using different approaches. The map from the current study, which was used as the input for Chromonomer at the second scaffolding step, was built using a custom implementation of the Lep-MAP3 pipeline, whereas the map from Sutherland et al. was produced with JoinMap4.

**Figure S2.** *k*-mer frequency spectrum of the raw PacBio reads used for generating draft contigs of the *S. fontinalis* genome assembly. *k*-mers frequencies were first computed using jellyfish *count* (--mer-len 21 --size 160M –canonical) and *histo*. The resulting .histo file was then uploaded to the web browser version of GenomeScope 1.0 using 10kb as the input read length to produce the plot. Observed *k*-mer frequencies (*y*-axis) relative to sequencing coverage (*x*-axis) are depicted in blue. Low-coverage *k*-mers attributable to sequencing errors are shown in orange. The black curve shows predicted *k*-mer frequencies without *k*-mers with sequencing errors, whereas the yellow curve shows *k*-mer frequencies of unique regions. Given that a highly homozygous doubled haploid sample was used, the main peak reflects the effective sequencing depth (~46X).

**Figure S3.** Distribution of copy number of each transcript of the Ssal_v3.1 transcriptome (GCF_905237065.1; 112,897 transcripts) in the brook trout assembly as well as in six other salmonid genome assemblies (see Table S4 for full assembly accessions).

**Supplementary tables**

**Table S1.** Mortality and microphthalmia ("pin-eyed" eggs) across treatment and control groups until week 8 following fertilization of eggs. Eggs from groups B, C and D were irradiated for 229, 458 (target dose) and 687 seconds, respectively, while group A eggs were fertilized with unirradiated milt. Egg groups 1 to 5 were shocked at 296, 316, 336, 356 and 376 minutes post-fertilization at 10,000 psi for 5 minutes. Eggs from the shock time group 0 were not shocked.

**Table S2.** Alevin mortality across shock treatment and control groups from weeks 8 to 15 after fertilization. Asterisks (*) indicate counts of individuals that were collected as eggs, not alevins.

**Table S3.** Assembly statistics for different salmonid reference genomes. Chromosome scale corresponds to the proportion of all assembly length that is anchored into the chromosomes. Asterisks (*) indicate that chromosome count includes mitochondria. ICSASG: International Cooperation to Sequence the Atlantic Salmon Genome

**Table S4.** Functional annotation of genomic features of various salmonid genome assemblies obtained from the NCBI Eukaryotic Annotation Pipeline. Total gene count excludes pseudogenes. BUSCO gene counts were obtained using BUSCO version 5.8.2 with the actinopterygii_odb10 lineage database (2024-01-08; 3,640 genes).

**Table S5.** Summary of BUSCO gene counts and mapped transcripts for the seven salmonid assemblies chosen for comparison. BUSCO version 5.8.2 was used with the actinopterygii_odb10 lineage database (2024-01-08; 3,640 genes). Values in parentheses next to gene counts denote the corresponding percentage of total 3,640 genes. The reference transcriptome used is from the Ssal_v3.1 assembly (GCF_905237065.1) and features 112,897 transcripts.

**Table S6.** Proportion of the length of each chromosome for which a homeologous region was found. Overlapping synteny block coordinates were first merged together with bedtools *merge* to obtain a set of intervals covered by a homeologous block in each chromosome. The total number of base pairs in these intervals was then divided by chromosome length.

**Table S7.** Coordinates of putatively collapsed regions in the brook trout assembly. These are regions where read coverage (estimated by 1-Mb windows) exceeds the sum of the mean genome-wide read depth plus twice the standard deviation and for which no homeologous region was found elsewhere in the assembly.

**Table S8.** Correspondence between linkage groups identified by Sutherland et al. (2016) and chromosomes in the brook trout genome assembly (ASM2944872v1; GenBank accession GCA_029448725.1).

**Supplementary data files**

**Supplementary Data file 1.** Description of the 11 microsatellite markers used for testing for homozygosity of putatively doubled haploid brook trout, including forward (F) and reverse (R) primer sequences.

**Supplementary Data file 2.** Genotypes of hatchery-reared brook trout at 11 microsatellite loci detailed in Supplementary Data file 1. Following salt extraction of DNA (Aljanabi & Martinez, 1997), PCR amplification of microsatellite was performed using Qiagen's Multiplex PCR kit (product no 206145; Amplification of Microsatellite Loci Using Multiplex PCR protocol). Three separate amplification reactions were conducted with a different set of primers to account for their different annealing temperatures (Reaction A: SfoB52, SfoC28, SfoC88, SfoC113, SfoC129; reaction B: SfoC24, SfoD75, SfoD100; reaction C: Sco216, Sco218, Sfo262Lav). Each reaction used 2 μL of input DNA, 5 μL of master mix, 2.4 μL of primer mix and 0.6 μL of RNAse-free water. All three reactions were conducted in 34 cycles of the following schedule, after an initial 15 minutes activation at 95°C and before a final extension period of 10 minutes at 72°C: denaturation for 30 seconds at 94°C, annealing at 60°C (reaction B) or 64°C (reactions A and C) for 3 minutes, and extension at 72°C for 1 minute.1 μL of PCR product was diluted in 10 μL of water, then 1 μl of this diluted solution was mixed into 10 μL of Hi-Di Formamide (Applied Biosystems) and loaded in a ABI3100 sequencer along with a GeneScan 500 ROX size standard. PCR fragments were visualized and scored using GENOTYPER 3.7. Three putatively haploid samples were in fact diploid (red alleles).

**Supplementary Data file 3.** State of each BUSCO gene (Complete, Duplicated, Fragmented, Missing) across all compared salmonid assemblies. BUSCO version 5.8.2 was used with the actinopterygii_odb10 lineage database (2024-01-08; 3,640 genes).

**Supplementary Data file 4.** Mapped copy number for each transcript of the Ssal_v3.1 transcriptome (GCF_905237065.1; 112,897 transcripts) across seven salmonid assemblies. Transcripts were mapped using GMAP (version 2019-03-15; Wu & Watanabe 2005). Only 'mRNA' entries of the GMAP output with a minimum sequence identity of 80% were considered successfully mapped. A count of 0 indicates a transcript that could not be mapped.
