# Supplementary material for: Chromosome-level genome assembly of a doubled haploid brook trout (Salvelinus fontinalis)
Source: G3 (Bethesda). 2025 Mar 25;15(6):jkaf066. doi: 10.1093/g3journal/jkaf066 (PMC12134987; doi:10.1093/g3journal/jkaf066)
Supplement: jkaf066_Supplementary_Data [file jkaf066_supplementary_data.zip › Table_S1_G3-2024-405170.docx]

**Table S1.** Mortality and microphthalmia (“pin-eyed” eggs) across treatment and control groups until week 8 following fertilization of eggs. Eggs from groups B, C and D were irradiated for 229, 458 (target dose) and 687 seconds, respectively, while group A eggs were fertilized with unirradiated milt. Egg groups 1 to 5 were shocked at 296, 316, 336, 356 and 376 minutes post-fertilization at 10,000 psi for 5 minutes. Eggs from the shock time group 0 were not shocked.

| **Treatment groups** | | **Fertilized eggs** |  | **Mortality** | | **Microphtalmic eggs** | |  | **Putative doubled haploids** | **Total remaining eggs** |
| --- | --- | --- | --- | --- | --- | --- | --- | --- | --- | --- |
| **Shock Time** | **UV irradiation** |  |  | **Count** | **Fraction of initial count (%)** | **Count** | **Fraction of initial count (%)** |  |  |  |
| 0 | A | 892 |  | 706 | 79.15 | 0 | 0.00 |  | - | 186 |
| 0 | B | 850 |  | 767 | 90.24 | 47 | 56.63 |  | - | 83 |
| 0 | C | 804 |  | 752 | 93.53 | 24 | 46.15 |  | - | 52 |
| 0 | D | 832 |  | 804 | 96.63 | 14 | 50.00 |  | - | 28 |
| 1 | B | 815 |  | 735 | 90.18 | 13 | 16.25 |  | 65 | 80 |
| 1 | C | 769 |  | 702 | 91.29 | 8 | 11.94 |  | 57 | 67 |
| 1 | D | 823 |  | 780 | 94.78 | 11 | 25.58 |  | 32 | 43 |
| 2 | B | 812 |  | 736 | 90.64 | 5 | 6.58 |  | 70 | 76 |
| 2 | C | 761 |  | 680 | 89.36 | 7 | 8.64 |  | 74 | 81 |
| 2 | D | 716 |  | 655 | 91.48 | 7 | 11.48 |  | 53 | 61 |
| 3 | B | 776 |  | 672 | 86.60 | 8 | 7.69 |  | 96 | 104 |
| 3 | C | 756 |  | 673 | 89.02 | 6 | 7.23 |  | 77 | 83 |
| 3 | D | 813 |  | 755 | 92.87 | 2 | 3.45 |  | 56 | 58 |
| 4 | B | 809 |  | 694 | 85.78 | 7 | 6.09 |  | 106 | 115 |
| 4 | C | 742 |  | 656 | 88.41 | 8 | 9.30 |  | 78 | 86 |
| 4 | D | 803 |  | 737 | 91.78 | 5 | 7.58 |  | 60 | 66 |
| 5 | B | 726 |  | 601 | 82.78 | 11 | 8.80 |  | 109 | 125 |
| 5 | C | 857 |  | 756 | 88.21 | 14 | 13.86 |  | 83 | 101 |
| 5 | D | 934 |  | 838 | 89.72 | 8 | 8.33 |  | 87 | 96 |
