# Supplementary material for: Chromosome-level genome assembly of a doubled haploid brook trout (Salvelinus fontinalis)
Source: G3 (Bethesda). 2025 Mar 25;15(6):jkaf066. doi: 10.1093/g3journal/jkaf066 (PMC12134987; doi:10.1093/g3journal/jkaf066)
Supplement: jkaf066_Supplementary_Data [file jkaf066_supplementary_data.zip › Table_S2_G3-2024-405170.docx]

**Table S2.** Alevin mortality across shock treatment and control groups from weeks 8 to 15 after fertilization. Asterisks (*) indicate counts of individuals that were collected as eggs, not alevins.

| **Treatment groups** | | **Initial egg count** |  | **Mortality** | |  | **Remaining eggs** |
| --- | --- | --- | --- | --- | --- | --- | --- |
| **Shock Time** | **UV irradiation** |  |  | **Count** | **Fraction of initial count (%)** |  |  |
| 1-2-3-4-5 | B-C-D (pin-eyed) | 205 |  | 190 | 92.68 |  | 15* |
| 1 | B-C-D | 154 |  | 53 | 34.42 |  | 101 |
| 2 | B-C-D | 197 |  | 62 | 31.47 |  | 135 |
| 3 | B-C-D | 229 |  | 74 | 32.31 |  | 155 |
| 4 | B-C-D | 244 |  | 69 | 28.28 |  | 175 |
| 5 | B-C-D | 279 |  | 72 | 25.81 |  | 207 |
| 0 | A | 186 |  | 3 | 1.61 |  | 183 |
| 0 | B | 83 |  | 72 | 86.75 |  | 11* |
| 0 | C | 52 |  | 43 | 82.69 |  | 9* |
| 0 | D | 28 |  | 23 | 82.14 |  | 5* |
