# Supplementary material for: Chromosome-level genome assembly of a doubled haploid brook trout (Salvelinus fontinalis)
Source: G3 (Bethesda). 2025 Mar 25;15(6):jkaf066. doi: 10.1093/g3journal/jkaf066 (PMC12134987; doi:10.1093/g3journal/jkaf066)
Supplement: jkaf066_Supplementary_Data [file jkaf066_supplementary_data.zip › Table_S3_G3-2024-405170.docx]

**Table S3.** Assembly statistics for different salmonid reference genomes. Chromosome scale corresponds to the proportion of all assembly length that is anchored into the chromosomes. Asterisks (*) indicate that chromosome count includes mitochondria. ICSASG: International Cooperation to Sequence the Atlantic Salmon Genome

|  | **Brook trout (*Salvelinus fontinalis*)** | **Lake trout (*Salvelinus namaycush*)** | ***Dolly Varden* (*Salvelinus* sp. IW2-2015)** | **Rainbow trout (*Oncorhynchus mykiss*)** | **River trout (*Salmo trutta*)** | **Atlantic salmon (*Salmo salar*)** | **Lake whitefish (*Coregonus clupeaformis*)** |
| --- | --- | --- | --- | --- | --- | --- | --- |
| Assembly name | ASM2944872v1 | SaNama_1.0 | ASM291031v2 | USDA_OmykA_1.1 | fSalTru1.1 | ICSASG_v2 | ASM2061545v1 |
| GenBank accession | GCA_029448725.1 | GCA_016432855.1 | GCA_002910315.2 | GCA_013265735.3 | GCA_901001165.1 | GCA_000233375.4 | GCA_020615455.1 |
| RefSeq accession | GCF_029448725.1 | GCF_016432855.1 | GCF_002910315.2 | GCF_013265735.2 | GCF_901001165.1 | GCF_000233375.1 | GCF_020615455.1 |
| Total length (bp) | 2,496,185,110 | 2,345,513,378 | 2,169,553,147 | 2,341,688,614 | 2,371,880,186 | 2,966,890,203 | 2,753,287,365 |
| Total number of scaffolds | 2,721 | 4,121 | 15,255 | 743 | 1,441 | 232,155 | 7,334 |
| N50 | 50,983,633 | 44,976,251 | 36,001,405 | 79,455,637 | 52,209,666 | 80,503,876 | 52,021,072 |
| L50 | 19 | 20 | 22 | 13 | 18 | 15 | 22 |
| N90 | 985,419 | 249,999 | 84,640 | 43,716,683 | 25,483,426 | 3,698 | 169,363 |
| L90 | 60 | 342 | 2,204 | 30 | 39 | 35,114 | 1,238 |
| Number of chromosomes | 42 | 43* | 40* | 33* | 41* | 30* | 41* |
| Chromosome scale | 0.889 | 0.845 | 0.699 | 0.954 | 0.915 | 0.755 | 0.725 |
| Longest scaffold length (bp) | 101,316,066 | 98,200,354 | 90,519,428 | 103,806,877 | 97,529,106 | 159,038,749 | 99,647,553 |
| Smallest scaffold length (bp) | 10,004 | 9,606 | 887 | 16,642 | 6,004 | 1,000 | 1,014 |
| Missingness | 0.002 | 0.000 | 0.000 | 0.003 | 0.031 | 0.117 | 0.000 |
| Gaps | 4,299 | 3,264 | 96,902 | 486 | 3,941 | 138,207 | 1,159 |
| GC content | 0.435 | 0.435 | 0.432 | 0.435 | 0.434 | 0.431 | 0.441 |
| Assembly method | Long reads 120X; Hi-C; short reads 70-80X | Long reads 89.0X; Hi-C; short reads | Long reads, short reads | Long reads 111.6X; optical mapping; Hi-C | Long reads 68X; single cell 70X; Hi-C 28X; optical mapping | Sanger 0.1-3.9X; Illumina mate pair 30.8-58.4X; Illumina paired end 9.1-66.8X; PacBio 19.2X | Long reads 20X |
| Reference | Current study | Smith et al. 2021 | Christensen et al. 2018b | Gao et al. 2021 | Hasen et al. 2021 | ICSASG 2015; Lien et al. 2016 | Université Laval 2021 |
