# Supplementary material for: Chromosome-level genome assembly of a doubled haploid brook trout (Salvelinus fontinalis)
Source: G3 (Bethesda). 2025 Mar 25;15(6):jkaf066. doi: 10.1093/g3journal/jkaf066 (PMC12134987; doi:10.1093/g3journal/jkaf066)
Supplement: jkaf066_Supplementary_Data [file jkaf066_supplementary_data.zip › Table_S4_G3-2024-405170.docx]

**Table S4.** Functional annotation of genomic features of various salmonid genome assemblies obtained from the NCBI Eukaryotic Annotation Pipeline. Total gene count excludes pseudogenes. BUSCO gene counts were obtained using BUSCO version 5.8.2 with the actinopterygii_odb10 lineage database (2024-01-08; 3,640 genes).

| **Annotated features** | **Brook trout (*Salvelinus fontinalis*)** | **Lake trout (*Salvelinus namaycush*)** | ***Dolly Varden* (*Salvelinus* sp. IW2-2015)** | **Rainbow trout (*Oncorhynchus mykiss*)** | **River trout (*Salmo trutta*)** | **Atlantic salmon (*Salmo salar*)** | **Lake whitefish (*Coregonus clupeaformis*)** |
| --- | --- | --- | --- | --- | --- | --- | --- |
| Assembly name | ASM2944872v1 | SaNama_1.0 | ASM291031v2 | USDA_OmykA_1.1 | fSalTru1.1 | ICSASG_v2 | ASM2061545v1 |
| GenBank accession | GCA_029448725.1 | GCA_016432855.1 | GCA_002910315.2 | GCA_013265735.3 | GCA_901001165.1 | GCA_000233375.4 | GCA_020615455.1 |
| RefSeq accession | GCF_029448725.1 | GCF_016432855.1 | GCF_002910315.2 | GCF_013265735.2 | GCF_901001165.1 | GCF_000233375.1 | GCF_020615455.1 |
| Number of genes | 56,058 | 46,242 | 42,343 | 69,903 | 58,157 | 55,227 | 55,237 |
| Average gene length (bp) | 23,670 | 21,163 | 20,856 | 18,975 | 21,446 | 21,207 | 22,422 |
| Number of protein-coding genes | 41,382 | 39,192 | 36,435 | 41,896 | 42,405 | 48,775 | 42,433 |
| Number of CDS | 83,302 | 58,230 | 59,939 | 97,744 | 87,841 | 97,546 | 76,885 |
| Average CDS length (bp) | 2,259 | 1,791 | 1,796 | 2,270 | 2,184 | 2,083 | 2,023 |
| Number of exons | 487,554 | 422,014 | 386,826 | 526,477 | 513,142 | 522,272 | 487,420 |
| Average exon length (bp) | 394 | 267 | 278 | 327 | 298 | 308 | 273 |
| Number of introns | 435,191 | 377,389 | 341,103 | 467,265 | 455,790 | 456,465 | 437,930 |
| Average intron length (bp) | 3,280 | 2,496 | 2,529 | 3,111 | 2,929 | 2,828 | 2,938 |
| Pipeline version | 10.1 | 8.5 | 8.0 | 8.5 | 8.2 | 9.0 | 9.0 |
|  |  |  |  |  |  |  |  |
| **BUSCO genes** |  |  |  |  |  |  |  |
| Complete and single copy (%) | 56.9 | 54.2 | 59.8 | 55.1 | 54.2 | 57.9 | 51.2 |
| Complete and duplicated (%) | 41.7 | 41.7 | 32.8 | 44.2 | 44.4 | 38.0 | 47.6 |
| *Complete (%)* | *98.6* | *95.9* | *92.6* | *99.4* | *98.6* | *96.0* | *98.7* |
| Fragmented (%) | 1.0 | 1.7 | 2.2 | 0.5 | 0.8 | 2.7 | 0.9 |
| Missing (%) | 0.4 | 2.4 | 5.2 | 0.2 | 0.6 | 1.4 | 0.4 |
