# Supplementary material for: Chromosome-level genome assembly of a doubled haploid brook trout (Salvelinus fontinalis)
Source: G3 (Bethesda). 2025 Mar 25;15(6):jkaf066. doi: 10.1093/g3journal/jkaf066 (PMC12134987; doi:10.1093/g3journal/jkaf066)
Supplement: jkaf066_Supplementary_Data [file jkaf066_supplementary_data.zip › Table_S5_G3-2024-405170.docx]

**Table S5.** Summary of BUSCO gene counts and mapped transcripts for the seven salmonid assemblies chosen for comparison. BUSCO version 5.8.2 was used with the actinopterygii_odb10 lineage database (2024-01-08; 3,640 genes). Values in parentheses next to gene counts denote the corresponding percentage of total 3,640 genes. The reference transcriptome used is from the Ssal_v3.1 assembly (GCF_905237065.1) and features 112,897 transcripts.

|  | **Brook trout (*Salvelinus fontinalis*)** | **Lake trout (*Salvelinus namaycush*)** | **Dolly Varden (*Salvelinus* sp. IW2-2015)** | **Rainbow trout (*Oncorhynchus mykiss*)** | **River trout (*Salmo trutta*)** | **Atlantic salmon (*Salmo salar*)** | **Lake whitefish (*Coregonus clupeaformis*)** |
| --- | --- | --- | --- | --- | --- | --- | --- |
| Assembly name | ASM2944872v1 | SaNama_1.0 | ASM291031v2 | USDA_OmykA_1.1 | fSalTru1.1 | ICSASG_v2 | ASM2061545v1 |
| GenBank accession | GCA_029448725.1 | GCA_016432855.1 | GCA_002910315.2 | GCA_013265735.3 | GCA_901001165.1 | GCA_000233375.4 | GCA_020615455.1 |
| RefSeq accession | GCF_029448725.1 | GCF_016432855.1 | GCF_002910315.2 | GCF_013265735.2 | GCF_901001165.1 | GCF_000233375.1 | GCF_020615455.1 |
| **BUSCO** |  |  |  |  |  |  |  |
| Complete and single copy | 2,071 (56.9) | 1,972 (54.2) | 2,177 (59.8) | 2,007 (55.1) | 1,973 (54.2) | 2,108 (57.9) | 1,863 (51.2) |
| Complete and duplicated | 1,517 (41.7) | 1,517 (41.7) | 1,194 (32.8) | 1,610 (44.2) | 1,616 (44.4) | 1,385 (38.0) | 1,731 (47.6) |
| *Complete* | *3,588 (98.6)* | *3,489 (95.9)* | *3,371 (92.6)* | *3,617 (99.4)* | *3,589 (98.6)* | *3,493 (96.0)* | *3,594 (98.7)* |
| Fragmented | 38 (1.0) | 63 (1.7) | 80 (2.2) | 17 (0.5) | 28 (0.8) | 97 (2.7) | 31 (0.9) |
| Missing | 14 (0.4) | 88 (2.4) | 189 (5.2) | 6 (0.2) | 23 (0.6) | 50 (1.4) | 15 (0.4) |
|  |  |  |  |  |  |  |  |
| **Transcripts** |  |  |  |  |  |  |  |
| Single copy | 94,419 | 93,646 | 91,129 | 92,459 | 98,142 | 97,707 | 87,411 |
| Duplicated | 16,369 | 16,615 | 17,713 | 18,069 | 13,620 | 14,586 | 21,155 |
| Missing | 2,109 | 2,636 | 4,055 | 2,369 | 1,135 | 604 | 4,331 |
