# Supplementary material for: Chromosome-level genome assembly of a doubled haploid brook trout (Salvelinus fontinalis)
Source: G3 (Bethesda). 2025 Mar 25;15(6):jkaf066. doi: 10.1093/g3journal/jkaf066 (PMC12134987; doi:10.1093/g3journal/jkaf066)
Supplement: jkaf066_Supplementary_Data [file jkaf066_supplementary_data.zip › Table_S6_G3-2024-405170.docx]

**Table S6.** Proportion of the length of each chromosome for which a homeologous region was found. Overlapping synteny block coordinates were first merged together with bedtools *merge* to obtain a set of intervals covered by a homeologous block in each chromosome. The total number of base pairs in these intervals was then divided by chromosome length.

| **Chromosome** | **Base pairs covered by an homeologous block (bp)** | **Total chromosome size (bp)** | **Proportion covered** |
| --- | --- | --- | --- |
| 1 | 101,316,066 | 79,020,555 | 0.78 |
| 2 | 101,148,069 | 80,744,988 | 0.80 |
| 3 | 78,939,564 | 75,767,938 | 0.96 |
| 4 | 88,210,620 | 66,028,239 | 0.75 |
| 5 | 67,316,613 | 60,001,629 | 0.89 |
| 6 | 86,004,203 | 70,233,241 | 0.82 |
| 7 | 69,297,716 | 56,663,882 | 0.82 |
| 8 | 52,002,740 | 46,172,586 | 0.89 |
| 9 | 61,936,799 | 55,741,201 | 0.90 |
| 10 | 50,268,153 | 44,594,315 | 0.89 |
| 11 | 60,085,304 | 45,613,337 | 0.76 |
| 12 | 60,416,927 | 56,547,196 | 0.94 |
| 13 | 55,592,301 | 54,332,310 | 0.98 |
| 14 | 56,307,559 | 50,914,778 | 0.90 |
| 15 | 47,696,033 | 47,134,505 | 0.99 |
| 16 | 53,561,650 | 51,181,772 | 0.96 |
| 17 | 48,822,140 | 45,230,441 | 0.93 |
| 18 | 62,731,796 | 60,547,245 | 0.97 |
| 19 | 56,108,426 | 49,916,358 | 0.89 |
| 20 | 46,220,702 | 43,182,440 | 0.93 |
| 21 | 52,113,258 | 47,193,343 | 0.91 |
| 22 | 42,625,803 | 42,403,309 | 0.99 |
| 23 | 50,983,633 | 46,678,115 | 0.92 |
| 24 | 46,851,096 | 43,616,480 | 0.93 |
| 25 | 43,302,116 | 40,460,359 | 0.93 |
| 26 | 48,152,825 | 41,379,505 | 0.86 |
| 27 | 45,259,495 | 41,385,993 | 0.91 |
| 28 | 50,913,443 | 45,235,407 | 0.89 |
| 29 | 46,201,713 | 39,810,226 | 0.86 |
| 30 | 42,718,502 | 35,792,060 | 0.84 |
| 31 | 49,840,808 | 43,046,263 | 0.86 |
| 32 | 45,901,657 | 45,848,705 | 1.00 |
| 33 | 51,265,593 | 43,335,562 | 0.85 |
| 34 | 39,882,818 | 24,915,577 | 0.62 |

**Supplementary Table 6 (cont.).** Proportion of length of each chromosome for which a homeologous region was found. Overlapping synteny block coordinates were first merged together with bedtools *merge* to obtain a set of intervals covered by a homeologous block in each chromosome. The total number of base pairs in these intervals was then divided by chromosome length.

| 35 | 43,061,335 | 28,660,414 | 0.67 |
| --- | --- | --- | --- |
| 36 | 34,452,196 | 30,574,089 | 0.89 |
| 37 | 34,569,483 | 33,431,575 | 0.97 |
| 38 | 36,714,413 | 30,013,848 | 0.82 |
| 39 | 28,793,718 | 18,903,445 | 0.66 |
| 40 | 30,609,635 | 14,135,601 | 0.46 |
| 41 | 24,386,083 | 23,256,416 | 0.95 |
| 42 | 26,002,104 | 13,309,547 | 0.51 |
