# Supplementary material for: Chromosome-level genome assembly of a doubled haploid brook trout (Salvelinus fontinalis)
Source: G3 (Bethesda). 2025 Mar 25;15(6):jkaf066. doi: 10.1093/g3journal/jkaf066 (PMC12134987; doi:10.1093/g3journal/jkaf066)
Supplement: jkaf066_Supplementary_Data [file jkaf066_supplementary_data.zip › Table_S7_G3-2024-405170.docx]

**Table S7.** Coordinates of putatively collapsed regions in the brook trout assembly. These are regions where read coverage (estimated by 1-Mb windows) exceeds the sum of the mean genome-wide read depth plus twice the standard deviation and for which no homeologous region was found elsewhere in the assembly.

| **Chromosome** | **Start** | **Stop** |
| --- | --- | --- |
| 4 | 88,000,000 | 88,210,620 |
| 19 | 55,000,000 | 56,000,000 |
| 19 | 56,000,000 | 56,108,426 |
| 21 | 52,000,000 | 52,113,258 |
| 26 | 48,000,000 | 48,152,825 |
| 34 | 34,000,000 | 35,000,000 |
| 34 | 39,000,000 | 39,882,818 |
| 40 | 0 | 1,000,000 |
| 40 | 1,000,000 | 2,000,000 |
| 40 | 4,000,000 | 5,000,000 |
| 40 | 5,000,000 | 6,000,000 |
| 40 | 12,000,000 | 13,000,000 |
| 40 | 13,000,000 | 14,000,000 |
| 40 | 30,000,000 | 30,609,635 |
