# Supplementary material for: Chromosome-level genome assembly of a doubled haploid brook trout (Salvelinus fontinalis)
Source: G3 (Bethesda). 2025 Mar 25;15(6):jkaf066. doi: 10.1093/g3journal/jkaf066 (PMC12134987; doi:10.1093/g3journal/jkaf066)
Supplement: jkaf066_Supplementary_Data [file jkaf066_supplementary_data.zip › Table_S8_G3-2024-405170.docx]

**Table S8.** Correspondence between linkage groups identified by Sutherland et al. (2016) and chromosomes in the brook trout genome assembly (ASM2944872v1; GenBank accession GCA_029448725.1).

| **Linkage groups from Sutherland et al. (2016)** | **Assembly chromosomes** |
| --- | --- |
| 1 | 1 |
| 8 | 2 |
| 3 | 3 |
| 6 | 4 |
| 4 | 5 |
| 7 | 6 |
| 5 | 7 |
| 18 | 8 |
| 15 | 9 |
| 10 | 10 |
| 2 | 11 |
| 20 | 12 |
| 9 | 13 |
| 17 | 14 |
| 12 | 15 |
| 35 | 16 |
| 22 | 17 |
| 21 | 18 |
| 26 | 19 |
| 19 | 20 |
| 13 | 21 |
| 23 | 22 |
| 24 | 23 |
| 25 | 24 |
| 16 | 25 |
| 11 | 26 |
| 31 | 27 |
| 40 | 28 |
| 33 | 29 |
| 27 | 30 |
| 30 | 31 |
| 34 | 32 |
| 38 | 33 |
| 14 | 34 |
| 28 | 35 |
| 32 | 36 |
| 36 | 37 |
| 29 | 38 |
| 37 | 39 |
| 42 | 40 |
| 39 | 41 |
| 41 | 42 |
